# Stabilization of Transfected Cells Expressing Low-Incidence Blood Group Antigens: Novel Methods Facilitating Their Use as Reagent-Cells

**DOI:** 10.1371/journal.pone.0161968

**Published:** 2016-09-07

**Authors:** Cecilia González, Rosa Esteban, Carme Canals, Eduardo Muñiz-Díaz, Núria Nogués

**Affiliations:** Immunohematology Laboratory, Banc de Sang i Teixits, Barcelona, Spain; Wake Forest Institute for Regenerative Medicine, UNITED STATES

## Abstract

**Background:**

The identification of erythrocyte antibodies in the serum of patients rely on panels of human red blood cells (RBCs), which coexpress many antigens and are not easily available for low-incidence blood group phenotypes. These problems have been addressed by generating cell lines expressing unique blood group antigens, which may be used as an alternative to human RBCs. However, the use of cell lines implies several drawbacks, like the requirement of cell culture facilities and the high cost of cryopreservation. The application of cell stabilization methods could facilitate their use as reagent cells in clinical laboratories.

**Methods:**

We generated stably-transfected cells expressing low-incidence blood group antigens (Di^a^ and Lu^a^). High-expresser clones were used to assess the effect of TransFix^®^ treatment and lyophilization as cell preservation methods. Cells were kept at 4°C and cell morphology, membrane permeability and antigenic properties were evaluated at several time-points after treatment.

**Results:**

TransFix^®^ addition to cell suspensions allows cell stabilization and proper antigen detection for at least 120 days, despite an increase in membrane permeability and a reduction in antigen expression levels. Lyophilized cells showed minor morphological changes and antigen expression levels were rather conserved at days 1, 15 and 120, indicating a high stability of the freeze-dried product. These stabilized cells have been proved to react specifically with human sera containing alloantibodies.

**Conclusions:**

Both stabilization methods allow long-term preservation of the transfected cells antigenic properties and may facilitate their distribution and use as reagent-cells expressing low-incidence antigens, overcoming the limited availability of such rare RBCs.

## Introduction

Antibodies against blood group antigens can induce clinical conditions such as haemolytic transfusion reactions, haemolytic disease of the fetus and newborn (HDFN) and autoimmune haemolytic anaemia. The detection and identification of blood group alloantibodies is therefore crucial in blood transfusion and in those pregnancies with fetomaternal incompatibility and risk of HDFN. Current antibody identification methods rely on panels of human red blood cells (RBCs) that have a limited viability and may carry biohazard risks. Besides, these panel RBCs simultaneously express a large number of antigens, which makes the antibody identification method to be based on the lack of reactivity with antigen-negative cells. This indirect determination of the antibody specificity is more complex when multiple antibodies are present in a patient’s serum. In addition to this, RBCs expressing low-incidence blood group antigens are not easily available, which hampers their inclusion in these panels. These problems have been addressed by generating cell lines stably expressing a unique RBC membrane protein, which may be used as reagent-cells to identify antibodies in the serum of sensitized patients. In this sense, several blood group proteins have been expressed in cells lines, like RhD/CE [1, 2], Kell [3, 4], Duffy [4, 5]; Kidd [6, 7], CR1 [4], Lutheran [8] and Band 3 [7, 9], and the recombinant antigens have been respectively recognized by specific antibody reagents.

Flow cytometric analysis of cell surface antigens requires, though, a cell treatment that preserves membrane integrity and causes minimal damage to the membrane proteins of interest. These features are met by fresh cells growing in culture. However, cell culture requires specialized laboratory equipment and trained personnel. Moreover, storage of cryopreserved cells in liquid nitrogen (N_2_) tanks or freezers also implies several drawbacks, such as a high cost, risk of transient warming events and low recovery during cell-thawing [10]. Furthermore, stable antigen expression in transfected cell lines is sometimes lost after many passages and repeated freezing and thawing.

The development of preservation methods other than cryopreservation could overcome some of these problems, allowing antigen stabilization, easy shipment and inexpensive storage, which would, in turn, facilitate the transfected cells application as reagent-cells in diagnostic laboratories.

Protocols to generate stabilized cells were initially developed for the evaluation of cytometer performance in different immunofluorescence assays [11, 12] and to permit transportation of whole blood specimens in sub-optimal conditions without inducing the morphological and phenotypical changes appearing in fresh blood samples, [13, 14]. In particular, a stabilization product called TransFix^®^ was shown to maintain cell integrity of whole blood specimens for at least 10 days, without affecting the accuracy of lymphocyte subset definition and their absolute cell count [13, 15–19]. TransFix^®^ is based on an aqueous solution containing paraformaldehyde and transition metals such as manganese and chromium [20].

Another interesting approach to stabilize mammalian cells is lyophilization or freeze drying. Important advances have been made in this field since it was first reported that small carbohydrates, found in high concentrations in many anhydrobiotic organisms, can stabilize cellular and macromolecular structures in the dry state [21, 22]. It has been shown that α,α-trehalose is particularly effective in preventing membrane damage upon drying in comparison to other sugar molecules [23–26]. This higher efficacy appears to involve the α,α-(1->1) glycosidic linkage of the two glucopyranose rings, allowing this molecule to adopt a clam shelf structure that facilitates interactions between the sugar and adjacent lipids [27]. Methods for the desiccation and dry storage of human cells (RBCs, platelets, stem cells, and gametes) are not still available for clinical applications [28, 29]. However, freeze-dried cells have been used for some diagnostic applications [30, 31] and as biological controls for cell counting in blood cell analyzers and for flow cytometry assays [32].

Here we describe the generation of transfectant clones stably expressing unique low-incidence blood group antigens. We used these cells as a model system to test stabilization methods, evaluating cell morphology, membrane integrity and antigen expression at different time points after the stabilization procedures. These methods could facilitate the use of such transfected cells in the investigation of erythrocyte antibodies.

## Material and Methods

### Serum samples

Non-commercial human sera containing anti-Di^a^ or anti-Lu^a^ antibodies were obtained from blood donations of alloimmunized blood donors. Written consent had been obtained prior to blood donation, in accordance with institutional guidelines. This written consent covers the potential use of blood components in biomedical research activities approved by clinical research ethics committees. The use of these serum samples in this study was approved by the Clinical Research Ethics Committee of the Vall d’Hebron Hospital (Ref: PRBST 103/2009).

### Generation of expression constructs

The cDNA corresponding to the erythrocyte membrane protein band 3 (*SLC4A1* gene, solute carrier family 4 anion exchanger member 1, Diego blood group, NCBI Reference Sequence: NG_007498.1) was kindly provided by David Anstee from the International Blood Group Reference Laboratory (IBGRL) in Bristol. This cDNA was modified to obtain the sequence encoding the low incidence Di^a^ antigen (c.2561C>T, p.Pro854Leu) using the QuickChangeTM Site-Directed Mutagenesis Kit (Stratagene, La Jolla, USA). A cassette including the Band 3 coding sequence, the internal ribosome entry site (IRES) sequence, and a green fluorescence protein (GFP)/neo gene (encoding a GFP fusion protein providing neomycin resistance) was subcloned into the pSF91 retroviral vector [33] to obtain the recombinant vector pSF91/Band 3-IRES-GFPneo.

The cDNA encoding the Lutheran blood group protein (*BCAM* gene, basal cell adhesion molecule, NCBI Reference Sequence: NG_007480.1) was obtained by reverse transcription using the SScriptIII Reverse Transcriptase (Invitrogen Corporation, Carlsbad, USA) from a whole blood RNA sample of an individual homozygous for the gene sequence encoding the high incidence Lu^b^ blood group antigen. This cDNA was amplified by PCR in two separate fragments. Primers 5’-ccgtgaacatggagcccccggacgcaccgg-3’ (nucleotides -8-22) and 5’-ataggtcccgctctggccccgggtcactcc-3’ (nucleotides 976–1005) were used to generate a 5’ LUTHERAN fragment of 1013 bp, and primers 5’-gacgagcgagactacgtgtg-3’ (nucleotides 355–374) and 5’-ttggctcagcactcgtctc-3’ (nucleotides 1874–1892) were used to generate a 3’LUTHERAN fragment of 1537 bp (for nucleotide numbering the A nucleotide of the ATG start codon was considered +1). The two amplification products have an overlapping sequence containing a XhoI restriction site. Each fragment was initially cloned into the pGEM vector and was then released by endonuclease digestion (the 5’ fragment with EcoRI and XhoI and the 3’ fragment with XhoI and SpeI). Subsequently, they were co-subcloned into pBS vector (digested with EcoRI and SpeI) as a complete cDNA sequence encoding the Lutheran protein. This cDNA was modified by site-directed mutagenesis (c.230G>A, p.Arg77His) to obtain the coding sequence of the Lu^a^ low-incidence variant. This sequence, as well as the most frequent counterpart encoding the Lu^b^ antigen, were subcloned into the mammalian expression vector pTurboRFP-N (Evrogen, Moscow, Russia), which was modified to introduce an IRES sequence between the gene of interest and the red fluorescence protein coding sequence (RFP).

### Generation of cell lines expressing blood group proteins

For Band 3 protein (Di^a^ antigen) expression we use the murine erythroleukemia (MEL) cell line [34], kindly provided by Débora Krimer from the Centro de Investigaciones Biomédicas (CBM) in Madrid, Spain.

The construct pSF/Band 3-IRES-GFPneo was introduced into the murine erythroleukemia (MEL) cell line by retroviral transduction [35]. Briefly, ecotropic NX-E packaging cells (kindly provided by G Nolan, Stanford University, Stanford, CA, USA) were transfected with the recombinant retroviral vector pSF91/Band 3-IRES-GFPneo by Transfectam reagent (Promega, Madison, USA). Filtered supernatants of the NX-E/ pSF91/Band 3-IRES-GFPneo cell line were used to infect MEL cell line. Neomicin resistant cells were selected with 800ug/mL (1.15mM) of G418 (Gibco-BRL, Life Technologies, Paisley, UK), and the cells expressing the fluorescent marker were isolated and cloned by cell sorting (FACSAria, BD Bioscience, San Jose, USA). The clones were then tested for expression of Di^a^ antigen by flow cytometry (see *Flow cytometric analysis*). Non-transfected MEL wild type cells (MELwt) were used as negative control.

Chinese hamster ovary (CHO-s) cells (GIBCO, Invitrogen Corporation, Carlsbad, USA, catalogue number 11619–012) were kept in adherent culture and were transfected with the pLua.Turbo.IRES.RFP and pLub.Turbo.IRES.RFP constructs. Briefly, 10uL of Transfectam reagent (Promega, Madison, USA) and 2ug of DNA were added to 60mm plates containing 70% confluent cells. After 72 hours, 800ug/mL (1.15mM) of G418 (Gibco-BRL) was added for the selection of the stably transfected cells. Resistant cells expressing the fluorescent marker were then isolated and cloned by cell sorting (FACSAria, BD Bioscience). Expression of the Lutheran protein in the different transfectant clones was also analyzed by flow cytometry (see *Flow cytometric analysis*), using CHO wild type cells (CHOwt) as negative control.

### Stabilization of transfected cells with TransFix^®^ reagent

TransFix^®^ reagent (Cytomark, Buckingham, UK) is intended to be added to whole blood samples at a 1/5 ratio (manufacturer instructions) to stabilize the leukocyte immunophenotype. To assess stabilizing properties of TransFix^®^ on transfected MEL and CHO cells, several reagent concentrations over a fixed cell concentration were tested. Cells growing in culture were washed twice with PBS and adjusted to 15x10^6^ cells/mL in PBS. TransFix^®^ reagent was added to cell suspensions at different ratios: 1/5, 1/10, 1/15 and 1/20, and each mixture was divided in several aliquots of 100 μL (1.5x10^6^ cells) and placed at 4°C for future analysis at different times (days 0, 1, 2, 3, 7, 10, 14, 28, 60, 90, and 120). The day of analysis, cells were rinsed with PBS, and cell morphology, membrane permeability, and antigen expression levels were evaluated by flow cytometry. Negative control cells (MELwt and CHOwt) were treated with TransFix^®^ in identical conditions.

### Trehalose treatment and lyophilization of transfected cell lines

Transfected MEL and CHO cells were suspended in cold PBS + 1% BSA, pelleted by centrifugation, and then resuspended in an isotonic solution containing either 0.3M or 0.6M of α,α-trehalose (Sigma-Aldrich, St Louis, USA). For each α,α-trehalose concentration, different temperatures (4°C, room temperature or 37°C) and incubation times (30, 60 or 90 minutes) were tested. After incubation, cells were pelleted and suspended in 0.3M of α,α-trehalose at 10x10^6^ cells/mL and divided in vials (1mL each). The vials were chilled at 4°C and placed at -80°C (at least for 2 hours) prior to lyophilization on a LYOBETA-35 device (Telstar, Terrassa, Spain). After a brief freezing step at -40°C, in order to stabilize chamber temperature, pressure was set to 0.05mBar and temperature to -33°C for primary drying. The end of the primary drying was determined by registering the product temperature variation (using a probe in a control vial). During secondary drying, temperature was set at 20°C and pressure was reduced below 0.01mBar (maximum vacuum). Once the freeze drying process was completed, the bottles were sealed under vacuum and kept at 4°C. The day of analysis, 1 ml of distilled water was added to each bottle. A fraction of the rehydrated cells (aprox. 1.5.10^6^ cells) was analyzed by flow cytometry at different times after lyophilization (24h., 15 days, and 4 months).

### Flow cytometric analysis

Membrane permeability, cell morphology and antigen expression levels in treated and fresh cells were evaluated by flow cytometry (FACScan, BD Bioscience) using the CellQuest Software (BD Bioscience). Membrane permeability was assessed by staining with 7-amino-actinomycin D (7-AAD, BD Bioscience). Forward and side scatter parameters (FSC and SSC) were used to evaluate cell morphology. Expression levels of the Di^a^ and Lu^a^ antigens were determined by indirect immunostaining and subsequent analysis of mean fluorescence intensity (MFI) values. Primary antibodies: Anti-Di^a^ monoclonal antibody (MoAb) HIRO.71 was kindly provided by Makoto Uchikawa, from the Tokyo Blood Center, and human polyclonal anti-Di^a^ sera were obtained from BioRad (Hercules, USA), Biotest (Dreieich, Germany), and from collaborators. Anti-Lutheran MoAbs, BRIC 221 and BRIC 224 were obtained from the IBGRL (Bristol, UK) and polyclonal anti-Lu^a^ sera were purchased from BioRad or available from our collection. Secondary antibodies: Fluorescein isothiocyanate (FITC)-conjugated Goat anti-mouse, (FITC)-conjugated Goat anti-human, and Phycoerytrin (PE)-conjugated Goat anti-human, were purchased from Jackson ImmunoResearch (West Grove, USA). Control cells for Di^a^ staining were MELwt cells immunostained in identical conditions, whilst CHOwt cells and/or CHO cells expressing the Lu^b^ antigen (CHOLub) were used as negative control for Lu^a^ staining.

### Statistical analysis

For each stabilization method, three independent experiments were performed (see S1 Statistics). Mean values and their standard deviations are presented. In order to obtain control cytometric parameters, fresh cells were also stained in matching conditions for analysis at each time point. Differences in MFI between treated and fresh cells were examined by the Paired Samples T-test (SPSS 15.0 for Windows). P values <0.05 were considered statistically significant.

## Results

### Di^a^ and Lu^a^ antigen expression in stably transfected cell lines

A cell line belonging to the erythroid lineage was needed for the expression of the Di^a^ antigen, as Band3 requires the coexpression of other erythroid specific membrane proteins (specially glycophorin A) for proper antigen expression on the cell membrane [9]. Therefore, we decided to use the MEL cell line. This murine erythroleukemia cell line would allow us, in addition, to decrease the likelihood of cross-reactions between endogenous and transfected blood group antigens. In contrast, CHO cells were selected for the Lutheran protein expression, as they are easily transfected and widely used for the expression of heterologous proteins. MEL clones expressing the Di^a^ construct and CHO clones expressing the Lu^a^ construct were generated and analyzed by flow cytometry as described in Materials and Methods.

**Fig 1:**
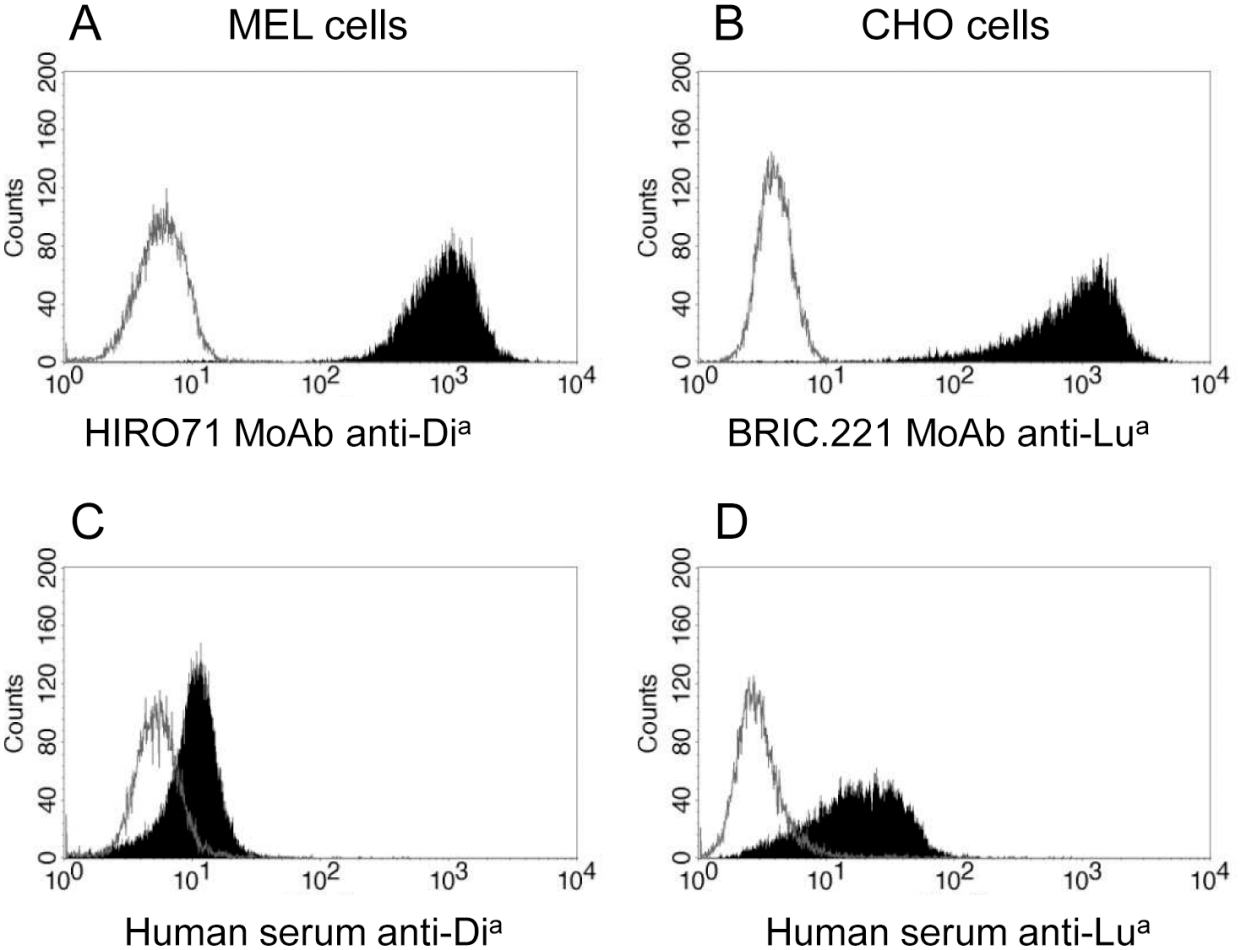
Antigen expression levels in high-expresser clones MELDia and CHOLua. **A.** Staining of MELDia.C7 cells with HIRO.71 anti-Di^a^ MoAb. **B.** Staining of CHOLua.B7 cells with BRIC.221 anti-Lutheran MoAb. **C.** Staining of MELDia.C7 cells with anti-Di^a^ human sera (DiaMed). **D.** Staining of CHOLua.B7 cells with human sera containing anti-Lu^a^ (from a sensitized patient). Gray empty histograms represent MELwt (A, C) or CHOwt (B, D) negative control cells stained in identical conditions.

Several clones were obtained for each transfection, and those that showed high levels of antigen expression and stability were expanded and cryopreserved. Clones MEL.Dia.C7 (Fig 1A) and CHO.Lua.B7 (Fig 1B) displaying high levels of the Di^a^ and Lu^a^ antigens, respectively, were selected for further studies. In addition to the antigen detection with MoAbs, we tested the reactivity of the transfected cells against a panel of known human sera of anti-Di^a^ or anti-Lu^a^ specificity. Specific binding reactions were observed with both, the MEL.Dia.C7 (Fig 1C, see also [7]) and the CHO.Lua.B7 cells (Fig 1D). In the Lua staining assays, we found similar negative reactions using CHOwt or CHOLub cells.

### Analysis of membrane permeability, cell morphology and antigen expression in preserved cells

#### Treatment with TransFix^®^ solution

As a first approach to evaluate the use of TransFix^®^ for the stabilization of transfected cells, a short-term experiment (28 days) was performed with MEL cells, using different reagent concentrations over a fixed cell concentration (15x10^6^ cells/mL in PBS). Based on the analysis of membrane permeability, cell morphology and antigen expression, the most favorable TransFix^®^/cell suspension ratio was found to be 1/15. This ratio was selected to perform further studies.

**Fig 2:**
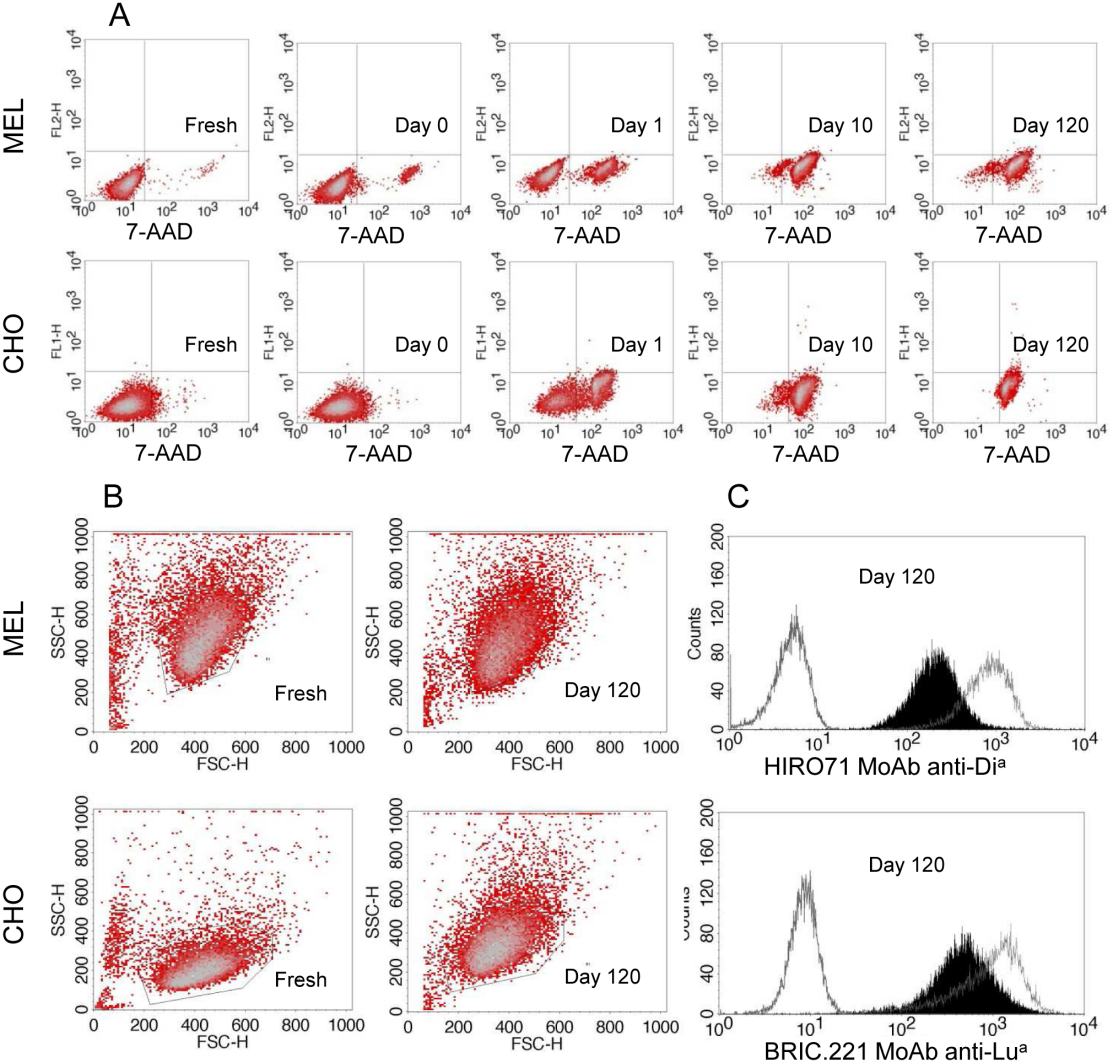
Analysis of membrane permeability, cell morphology and antigen expression in TransFix^®^ treated cells. **A.** Staining with 7-AAD reagent at different times after treatment and comparison with equivalent fresh cells. **B.** Forward (FSC) and side scatter (SSC) parameters were acquired at several time points (shown for 120 days), and compared with fresh cells. **C.** Antigen expression levels of MELDia cells (HIRO.71 staining) or CHOLua cells (BRIC221 staining) at 120 days of treatment (fresh cells are overlaid); negative controls were MELwt or CHOwt cells stained in matching conditions.

The first day after treatment (day 0), both MEL and CHO cells were negative for 7-AAD staining, as were the control cells, indicating an unaltered membrane permeability. At day 1, about 30% (MEL) or 50% (CHO) of cells (Table 1) were positive for 7-AAD staining, whilst after 8–10 days, all the cells were 7-AAD positive with a more homogeneous staining (Fig 2A and Table 1).

**Table 1:** Analysis of membrane permeability, cell morphology and antigen expression levels in TransFix^®^ preserved cells.

	MEL cells				CHO cells			
	7-AAD^a^	FSC	SSC	MFI^b^ ^HIRO.71MoAb^	7-AAD^a^	FSC	SSC	MFI^b^ ^BRIC221MoAb^
Fresh	2±1	408±8	418±7	100	4±3	406±15	229±15	100
Day 0	4±2	392±8	434±20	79±4	2±1	401±32	305±11	95±2
Day 1	32±6	389±8	445±45	55±22	48±14	395±16	306±12	84±7
Day 2	85±9	385±26	476±32	61±8	81±6	391±19	305±15	67±9
Day 3	95±4	389±15	499±36	67±6	84±5	406±40	308±15	58±3
Day 7	96±2	394±7	467±14	59±20	85±8	413±33	334±44	59±6
Day 10	97±2	394±8	464±13	46±17	87±15	419±33	337±34	57±11
Day 14	96±3	403±6	451±12	55±12	90±5	415±35	334±41	54±8
Day 28	94±3	398±13	447±13	49±6	91±1	409±26	322±10	54±6
Day 60	97±2	405±26	454±13	46±8	93±2	400±25	324±26	46±12
Day 90	98±2	406±18	461±14	38±8	96±2	397±28	330±13	52±7
Day 120	94±7	403±15	462±16	36±5	94±3	392±34	335±22	47±9

Data are expressed as Mean±SD (n = 3). FSC: forward scatter; SSC: side scatter; MFI: mean fluorescence intensity.

^*a*^ Percentage of cells positively stained with 7-AAD.

^*b*^ Expressed as a percentage of MFI for fresh cells (considered 100%).

A visible decrease in FSC was observed during the first days after TransFix^®^ treatment, both in MEL and CHO cells, although this parameter progressively tends to return to the initial values. In contrast, we detected mild increments in SSC in both cell lines, compared to fresh cells (Table 1). Importantly, both stabilized cell lines were observed as a unique cluster over all time periods (Fig 2B).

A progressive reduction in antigen expression levels over time was found in treated MEL and CHO cells, compared to fresh cells (p<0.001 for both cell lines). For better visualization, MFI values (HIRO.71 MoAb staining for MEL cells and BRIC221 MoAb staining for CHO cells) are expressed as a percentage of the MFI values of fresh untreated cells (assumed to have 100%). As it is shown in Table 1, MFI values after TransFix^®^ treatment ranged between 79±4% (Day 0) and 36±4% (Day 120) in the case of MEL cells, and between 95±15% (Day 0) and 47±9% (Day 120) in the case of CHO cells. Despite this reduction, the results obtained after 120 days of TransFix^®^ treatment show reproducible antigen detection on the transfected cell membrane (Fig 2C) with the monoclonal antibodies as well as with the human sera tested.

#### Lyophilization of α,α-trehalose-treated cells

**Fig 3:**
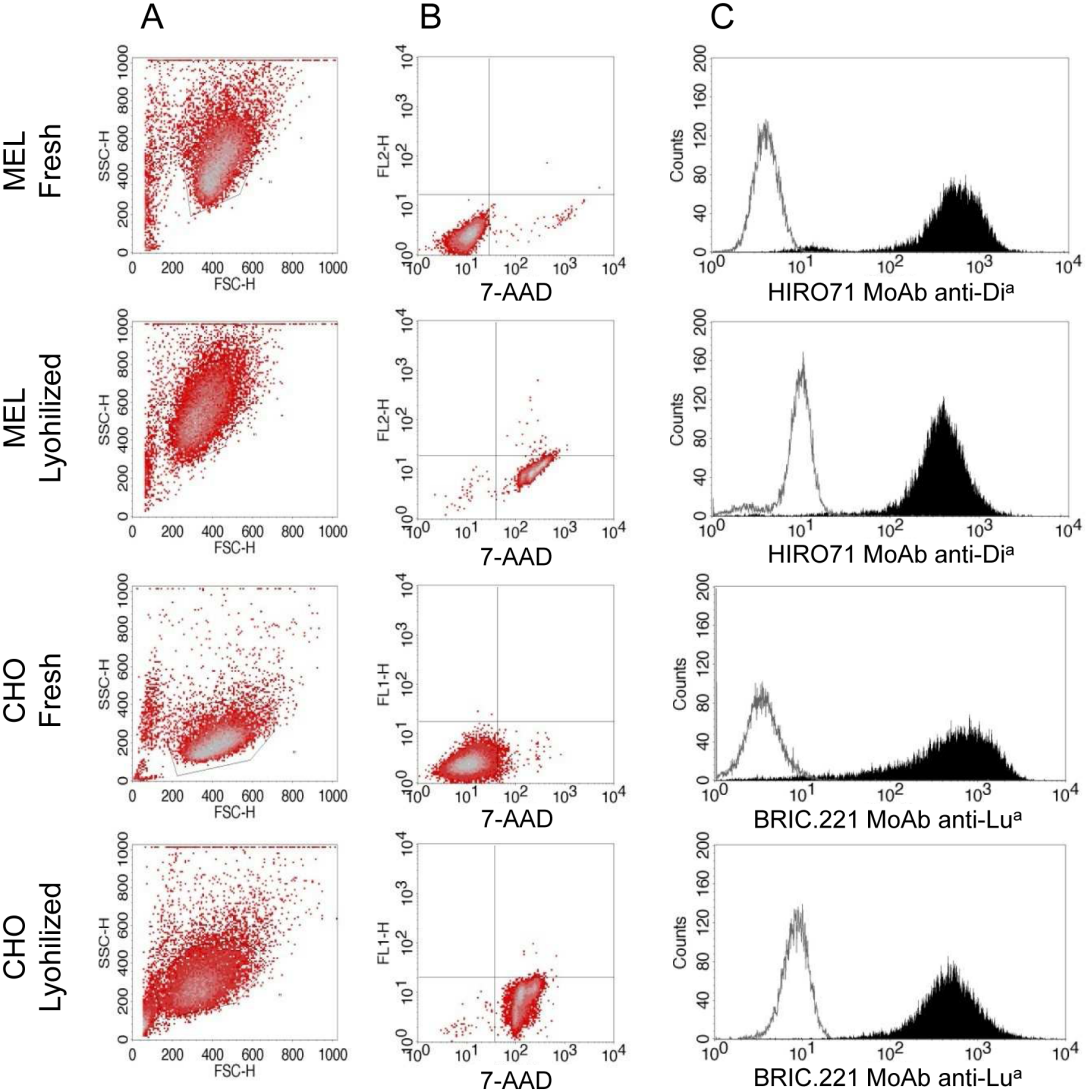
Analysis of cell morphology, membrane permeability and antigen expression levels in lyophilized MEL and CHO cells. **A.** Acquisition of forward (FSC) and side scatter (SSC) parameters. **B.** Staining with 7-AAD reagent. **C.** Antigen expression levels of rehydrated MELDia (HIRO.71 staining) or CHOLua cells (BRIC221 staining); negative controls were MELwt or CHOwt cells stained in matching conditions. All parameters were also analyzed for equivalent fresh cells for comparison.

Pretreatment with α,α-trehalose has been proven to play an important role in cell resistance to freeze-drying [23–26, 36]. In order to evaluate different pretreatment conditions, several α,α-trehalose concentration (0.3M or 0.6M), temperatures (4°C, room temperature, 37°C) and incubation times (30, 60 or 90 minutes) were assessed in a preliminary study with MEL cells. Lyophilized MEL cells were reconstituted in 1mL of distilled water and analyzed as described in methods. Incubation with 0.6M of α,α-trehalose at 37°C during 90 minutes were the conditions yielding a higher degree of conservation of membrane permeability, cell morphology and antigen expression after lyophilization, and these settings were used to perform further studies. Both, MEL and CHO cells, showed a moderate reduction in FSC and a mild increment in SSC parameters as compared with equivalent fresh cells (Fig 3A and Table 2). Lyophilized cells also have greater membrane permeability, as were positively stained with 7-AAD (Fig 3B and Table 2). Antigen expression levels in MEL cells were assessed with HIRO.71 MoAb, whilst CHO cells were stained with BRIC.221 MoAb. Although a reduction in MFI values was observed after the freeze-drying procedure in both cell lines (p<0.05), the level of antigen expression was rather conserved, in the range of 90% of the values detected with fresh cells (Fig 3C and Table 2). Thus, detection of the Di^a^ and Lu^a^ antigens is feasible in reconstituted cells after lyophilization. All parameters studied (morphology, permeability and antigen expression) were very similar for cells reconstituted at days 1, 15 or 120 after lyophilization (See coefficients of variation in Table 2), indicating a high stability of the freeze-dried product.

**Table 2:** Analysis of membrane permeability, cell morphology and antigen expression levels in freeze-dried cells.

	MEL cells				CHO cells			
	7-AAD^a^	FSC	SSC	MFI^b^ ^*HIRO*.*71MoAb*^	7-AAD^a^	FSC	SSC	MFI^b^ ^*BRIC221MoAb*^
Fresh	2±1	408±8	418±7	100	4±3	422±14	229±15	100
Day 1	97±3	339±27	521±13	90±2	97±3	364±47	298±37	90±4
Day 15	98±3	328±28	523±22	94±5	98±3	351±29	296±51	83±4
Day 120	98±2	326±26	530±26	89±5	96±3	340±18	299±41	91±8
CV^c^	0.9	2.1	0.9	2.6	0.9	3.4	0.4	5.1

Data are expressed as Mean±SD (n = 3) FSC: forward scatter; SSC: side scatter; MFI: mean fluorescence intensity. CV: coefficient of variation.

^*a*^ Percentage of cells positively stained with 7-AAD.

^*b*^ Expressed as a percentage of MFI for fresh cells (considered 100%).

^c^ For each parameter, CV between days 1, 15 and 120 is calculated as SD/Mean*100.

### Reactivity of human sera with stabilized transfected cells

**Fig 4:**
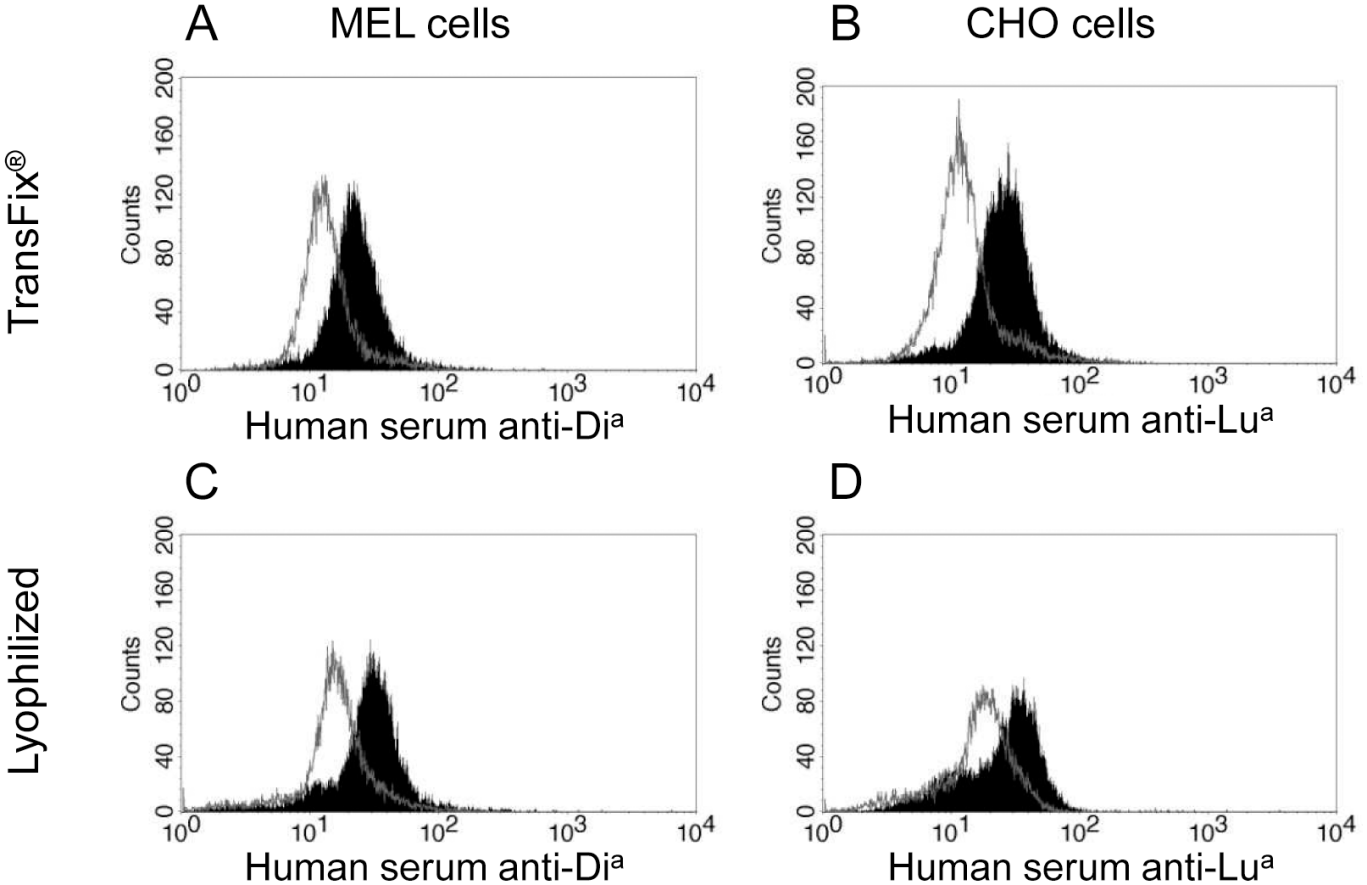
Reactivity of human sera against stabilized transfected cells. Human sera containing anti-Di^a^ or anti-Lu^a^ (from sensitized patients) were tested against TransFix^®^ treated, or lyophilized MELDia and CHOLua cells. Negative controls were MELwt or CHOwt cells stained in identical conditions (gray empty histograms).

The stabilized transfected cells were also tested against known human sera containing alloantibodies of anti-Di^a^ or anti-Lu^a^ specificity. Anti-Di^a^ specific binding was detected in both, TransFix^®^-treated (Fig 4A) and lyophilized (Fig 4C) MELDia cells. Likewise, anti-Lu^a^ binding reactions with TransFix^®^-treated (Fig 4B) and lyophilized (Fig 4D) cells could be clearly distinguished from reactions against stabilized CHOwt negative cells. Together, these results indicate that these stabilized transfected cells could potentially be used as reagent-cells to detect anti-Di^a^ and anti-Lu^a^ antibodies in patient’s sera.

## Discussion

Successful expression of the low incidence Di^a^ and Lu^a^ antigens has been obtained in two different mammalian heterologous systems: a murine erythroleukemia (MEL) cell line and a Chinese hamster ovary (CHO) cell line. Initial flow cytometry assays had shown specific detection of the recombinant antigen in the transfected cells membrane using both monoclonal and policlonal reagents [7]. In order to facilitate the use of these transfected cells as reagent-cells for erythroid antibody identification, we evaluated stabilization methods allowing long-term preservation of their antigenic properties. We first investigated several methods that have been previously used for cell stabilization, such as addition of isotonic nutrient solutions (commonly employed for panel RBCs stabilization) and chemical fixing reagents. However, transfected cell suspensions in such solutions were not suitable for antigen detection after 6 days at 4°C (data not shown). It had also been reported that the TransFix^®^ reagent stabilizes leukocytes in blood samples for at least 10 days [17, 19] without producing sample artifacts as other cell fixation reagents. Here, we demonstrated that this method can be adapted to stabilize transfected MEL and CHO cell lines. We tested several ratios of reagent to cell suspension (fixing the cell concentration at 15x10^6^ cells/mL in PBS) and found the 1/15 proportion to be optimal, allowing cell stabilization for at least 120 days. An increase in membrane permeability was observed in both cell lines after treatment with TransFix^®^. The variations in membrane permeability, however, are not associated with alterations of cell morphology, as TransFix^®^ treated cells were observed as a uniform cluster over all time periods. The stabilized cells showed a gradual decrease in antigen expression levels, but even at day 120 Di^a^ and Lu^a^ antigens were properly detected with the monoclonal antibodies and human sera tested. We cannot exclude, though, that low titer antibodies present in some human sera are not detected when using these stabilized reagent cells.

We also explored lyophilization as an interesting preservation method. It has been described that certain levels of intracellular α,α-trehalose are needed to protect cell membranes during freeze-drying [24, 25, 36], but introduction of the sugar into the cell is not simple and fluctuate depending on the cell type [25, 37]. Other variables such as freezing rate [38, 39] and temperature during sublimation [40] must be carefully controlled in order to preserve cell integrity. We worked on the hypothesis that, as a method to preserve reagent-cells, lyophilization is far simpler that mammalian cell desiccation for clinical applications. Firstly, it is not mandatory that cells retain all physiologic properties and, second, nonuniform drying is prevented as there is no need to process large volumes. We tested a simple protocol in which the cells are incubated in an isotonic solution containing α,α-trehalose as unique additive, without measuring intracellular trehalose concentrations. The cells were then put at -80°C (without controlling the freezing rate) and lyophilized under standard conditions (not at extreme low temperatures).

Although increments in membrane permeability (positive staining with 7-AAD) and morphologic variations are observed after lyophilization, antigen expression levels were comparable to those of the corresponding fresh cells. As mentioned earlier, intracellular levels of α,α-trehalose were not determined. However, lyophilized cells that had been previously treated with α,α-trehalose under conditions that presumably favors the sugar uptake [41]: at higher trehalose concentrations (0.6M), at higher temperatures (37°C) and during longer periods of time (90 minutes) were better preserved than lyophilized cells treated with 0.3M trehalose at 4°C and during 30 minutes, suggesting that superior membrane protective effects are achieved with a higher level of intracellular trehalose.

In conclusion, our data indicate that both, treatment of cells with TransFix^®^ reagent and lyophilization of trehalose treated cells, are valid methods allowing stabilization of the transfected cells. Several advantages are derived from these stabilization methods. First, the availability of ready to use vials of reagent-cells compared to maintaining the cells in culture till the day of analysis. Second, cell machinery is arrested after treatment with TransFix^®^ or after lyophilization, preventing the decrease of antigen expression levels that is frequently observed after freeze-thawing cycles. Lastly, stabilized cells can be stored and shipped at lower cost and risk, as compared to cryopreserved cells.

Due to the limited availability of cells positive for low-incidence blood group antigens, standard RBC panels usually do not include such reagent-cells. Overall, the cell stabilization methods optimized and evaluated in this study may facilitate the distribution and use of transfected cells expressing low-incidence antigens, which could supplement and improve RBC-based antibody identification assays. Likewise, these methods may potentially simplify the detection of alloantibodies against human neutrophil antigens (HNA) [42, 43] or human platelet antigens (HPA) [44, 45] by means of HNA and HPA transfected cells, also available.

## Supporting Information

S1 Statistics Individual data points for the three different TransFix and lyophilization experiments. (XLSX)
